# Take of the Leaven of Damnation

**Published:** 1880-01

**Authors:** Ben. H. Pratt


### THE BISTOURY.

Vol. XV. ELMIRA, N. Y., JANUARY, 1880. No. 4.

### Contributions.

### TAKE OF THE LEAVEN OF DAMNATION.

BEN. H. PRATT.

How few homes are happy homes for children. How many homes are the dreariest spots they are compelled to pass any portion of their lives in. How few parents know how to make their homes fit for children to dwell in, and fewer still are they who, knowing what a home should be, try to make it such. I do not ask that these statements be taken for granted, but I do ask that each one who may take the trouble to read these lines, would seek to disprove my assertion by testing the matter for himself. Look, my friend, at your own home. You may never have suspected that it is not a happy one for your children, if you are blest with these gifts. It may never have occurred to you, nor to her, who jointly with you has assisted in building up the home of which you are the head, that your hearth is not one at which the children find any cheer—that your table is not one around which it is your children's chief delight to gather. I may have made a mistake in your case, but if so you are one of a hundred —may be, one of a thousand. Having satisfied yourself upon this point, look further. Whether you dwell in the populous and stirring city, or within the quieter borders of a village, or in the still greater solitude of a sparsely settled region, go out among those of your acquaintance and carefully note the adaptation of the homes you find to the real happiness of the young who constitute the larger portion of the family circle you enter. Do this, and then, if you can, gainsay my opening assertion, namely: few indeed are the homes that are really fit to rear children in.

In this particular no distinction can be drawn between the homes of the rich and those of the poor. The homes of wealth are as widely open to criticism in this respect as are those of the middle and poorer classes. Money does not make happy homes, nor the lack of it unhappy ones, other things being equal. The palatial drawing room is drearier by reason of its gorgeousness, and the plain cottage is more dismal by reason of its bareness, if there be not within the mansion and the cottage something more than wealth can bestow, and something more than poverty can extract. Let no one excuse his unhomelike home because he is too hampered by business or social relations to give it his attention; nor yet let another claim that he has not the wherewithal to make his home glad. This is one of those matters into which neither wealth nor poverty enters as an important factor. Home is what we choose to make it, be our circumstances what they may, for happiness cannot be enticed within our houses by a glittering price, nor can it be bribed to desert the homeliest spot for a more brilliant abode. We are the arbiters of our own homes. What we make them, they are.

Our children are samples of our homes. As our homes are so will our children be. Show me a child that does not love its home better than any other place on earth, and I will show you a home that needs remodeling. Children are wiser than we know. Their powers of discernment are keener than our own. The mere infant of two years has a better knowledge of the defects in a home than its parents have. We are apt to think that if we provide shelter, food, clothing, care and education for our little ones, that we are doing our whole duty in the matter. We plume ourselves greatly when we go beyond these points and furnish our children with now and then a bauble of a plaything, and give them sole dominion over it. We do this oftener with a selfish than a benevolent motive. We provide them with what will enable them to take some of their care off from our hands, and give us surcease of nursing, or enable us to pursue our business or our pleasures uninterrupted by the teasings and plaints of our children. When they are older grown, we almost entirely give them over to their own devices, restricting them only by the proprieties, or what we choose to consider to be such. Arrived at still maturer years, we are prone to regard them as in need of no different enjoyments than those which we esteem sufficient for our own contentment. The father, upon an evening, buries himself in his newspaper or book, and the mother occupies herself with her sewing or other work, and the household must be mute. The youngsters must cease their romp and sit properly mum through the evening's hours. No questions must be asked, and no interruptions of the family quiet can be tolerated. By dint of long custom the children grow into a reconciliation with their surroundings, and however unhappy, begin to learn that there is no release from their thraldom, at least for a time. Children are not idiots. They know when an injustice is being done them. While you are absorbed in that which gives you pleasure, they are muttering unkind things of you in their thoughts, and long ago have taken the exact measure of your selfishness, and the complete dimensions of your meanness. With nothing left them but unlimited time to ponder upon these things, they ponder to some purpose. They fully realize the situation. They mean to endure it just so long as they are obliged to, and not one moment longer. The home is simply a temporary place to exist in, because they have no other. They know of homes where children are allowed some latitude. They have perhaps spent stolen hours there. They compare their lot with that of others and actually sigh for the freedom of the street urchin who, though unprovided for and uncared for, has at least the liberty to enjoy himself as far as his circumstances will allow. To get away from such a home for any length of time, however brief, is a joy unspeakable. Anywhere, anything, but that dreary home and that dismal evening of restraint. How can children become attached to such a home? What is to prevent them from fleeing from it when they are old enough to do so? Who shall blame them when they go out of it and by reason of such restraints they go to the other extreme of excess in all that has been denied them? Is it any wonder that boys, as soon as released from such a home, seek in the hotels and the saloons and the brothels the fullest freedom these places afford? Is it to be wondered at that girls, old enough to run abroad among their mates, conjure up all manner of forbidden enjoyments to recompense them for all the years of punishment they have undergone? They are only giving full fruition to the sensitive plant that parental neglect has distorted into what nature never meant it should be, and which it could never have been under proper cultivation.

It is a matter of constant wonder in society, why is it that boys and girls, reared in so-called model homes, go so quickly to the bad when they leave those homes. It should be no cause of wonderment—it is no wonder. The laws of compensation which we all hold to be so absolute in a physical sense, is no less absolute in the moral. You cannot exert a pressure upon material objects in one direction without at the same time finding a corresponding yielding in another direction. You cannot lay heavy restraints upon the natural ebullition of youth without discovering, to your sorrow, that there has been a reaction in the opposite direction. The harder a bow is bent, with so much greater force will it restore itself. The higher you lift a body from the earth, the swifter it descends thereto. The stricter you bind down your children to an unnatural propriety, the further from that propriety will they rebound when the thongs are severed. Your home is your children's home as well. As component parts of the family whole, they have their rights in that family; they are not interlopers. You brought them into your home, and by so doing gave them an inherent right to have the same joy of it that you have. But your pleasures are not theirs, at all times, and theirs are not yours. It is meet that each make some sacrifice, and not that one make all. You, by your might, assume the right to dictate what they shall do and shall not do, but they in their weakness can assume only what you will that they may. The result is your comfort for a time, and your sorrow for an eternity.

The child has rights in a home which the parent is bound to respect. It has a right to indulge its natural tendency to romp, to play, to be amused. You have a right to place bounds to any excess in these, but no more right to interdict them wholly, than you have to stop their breath. It is not only a cruel thing to do, but it is a dangerous thing. It is easy to crush the spirit from out young hearts, and there are parents who are doing it every day without realizing it. In the presence of father and mother there are children now sitting with bated breath lest they disturb papa or annoy mamma. Should one of these have the temerity to ask a question of either parent, the child is met with such a frown and such a rebuke as will rankle for aye in its tender heart. No, that is not a happy home where the voices of the children are hushed, and the rattle of their playthings and the rustle of their book-leaves evoke a marked sign of annoyance from their elders. I do not mean that loquacity, and chatter, and din, and romp, are to be allowed to run riot in the home; nor yet, that the young are to be encouraged in their forwardness and boldness of approach toward those older than they; nor, that they are to be given entire control of the home, and allowed to sway its inmates to their will. But their interests and their joys are to be consulted, and their share of the home's pleasures is to be meted out evenly to them, giving them rather an excessive measure than a scant one.

The parent is too often unwilling to be his child's companion in those amusements which he can adapt himself to if he will. He will not put himself upon their level at four, six and ten years of age, yet he will coo to, and trot and dandle and even wash and dress the baby of as many months. As soon as children begin to reason, and thereby approach somewhat nearer to the parental mind, for some reason I never could understand, they are regarded as fit subjects to be curbed and suppressed, and the breach between child and parent, which ought to lessen year by year, grows wider and wider, even to estrangement, then awe, then fear, then actual dislike; and when this point is reached there is no retracing of steps, nor any after restoration of the relations that should have become more and more intimate from birth to the period of a natural severance of the ties that normally bind the offspring to the authors of its being.

If parents would but take a common-sense view of the duties devolving upon them; if they would only put themselves in their children's place; if they would only try to recall their own feelings when they were children; if they would only sit down and reflect for a time upon the nature of childhood, and try to reason out the wherefore of all the estrangements they find between children and parents; if they would only determine to get at the cause of so many young people's aversion to their homes, and their infatuation for that which is wrong; if they would do this, it seems to me that any parent, with a modicum of sense, could discover the secret of that over which so many profess to wonder so amazedly. Such meditation, when boiled down, will result in something like this:

"That boy doesn't have a good time at home—never had a good time there—is bound to have it somewhere—if he can't have it at his own home he'll have it at somebody's else home, or wherever he can get it; that girl never was allowed any freedom to say or do as nature intended she should, and having been under pressure all her life, has concluded to get out of the toils somehow, and in her desperation has gone to the other extreme too far for reclamation. Had she been allowed more freedom at home; had he been made more of an equal in the household; had they been treated more like fellow beings and less like subjects, this trouble would never have occurred. Here is the rock on which so many have been wrecked. I see it—I can avoid it—I will."

What shall we do then ? We must first divest ourselves of our conceit, and come down to our children's level. We must next jerk off that straight-jacket of fear lest we cultivate in our children a taste for that which will, in after life, be harmful. We must not reason so narrowly as to conclude that if our children play cards at home it will create a taste for the gaming table; or if they dance at home they will resort to the dance-house; or if they indulge in charades and plays, or learn to sing well or recite well, or declaim well, that these will cultivate a taste for theatres and dissipation of other sort. We must reason broader than this. We must take for our premise the daily demonstrated fact that in ninteen cases out of twenty, boys and girls will learn to play cards, to dance, to recite, to sport, to sing, and to master countless other accomplishments not enumerated in the Westminster catechism; for others, with whom they associate, do these things, and why should not they? Now, whether is it better that they do these things at home, beneath your own roof, under your own eye, surrounded by all the refining and endearing influences of home and friends, or that they steal off at every opportunity and clandestinely and surreptitiously acquire the same accomplishments, you know not where, you know not when, nor within what baneful surroundings ? This is the way we must reason it, and when our much shocked neighbor, the deacon, calls and wants to know whether we are aware that we are heaping loads of damnation upon our children, ask him the above questions, and if he still wags his saintly head in grim doubt, tell him to send his children to inebriate asylums and magdalen homes if he wants to, but that you propose, sustained by a sense of duty and large common sense, to relieve the asylum and the magdalen shop of taking charge of your boys and girls when they grow up. Tell Mr. Straightlace that you will set up your theory against his, and begin as soon as he likes; that the test of the matter lies in the results obtained; that you will compare the reputations of your children with his at the end of ten or fifteen years and abide by the result.

The compression and seclusion and anti-fun plan would be all very well if there were no evil in the world; but there is such an appalling leaven of damnation all about our homes, that some of it must get into our children's minds and leaven the whole being. Now, whether is it not better, while this poison must enter into the minds of our children anyhow, we should prepare them for it and meet it upon the similia similibus curantur principle. Let us give them little doses of it ourselves, and treat them with antidotes at the same time. Let us vaccinate them with a little of the virus, so that they may have the disease in a milder form. Let us give them a taste of the forbidden fruit, lest in the fascination that comes from stealing it, they indulge to an excess and die a moral death. Let them see that we do not fear the enemy of their virtue, by taking our hand at cards with them—by joining in the dance with them —by going to the play with them—by encouraging their levity at times—but doing it becomingly, so that when they meet with the bedizenments of the outer world they may not be dazzled thereby, and become victims of their infatuation. Let them find no attractiveness in the novelty of these things, but rather discover that they are only the same they have long known, though exhibited in a more dangerous light. Thus, I believe, our children can be in a measure saved from the fascinating toils of what would otherwise prove new and therefore more seductive evils. Let us make our homes attractive for our children—the most attractive spots on earth. This will do more to keep them away from the temptations that beset youth on every hand, and do it more easily and more effectually, and redound more to their well being here and hereafter, than all the constraint and severity that ever were exercised in the homes of those who mistakenly pride themselves upon their knowledge of the way in which children should be taught to go.

